# Commentary: Memory CD8^+^ T cells colocalize with IL-7^+^ stromal cells in bone marrow and rest in terms of proliferation and transcription

**DOI:** 10.3389/fimmu.2016.00102

**Published:** 2016-03-31

**Authors:** Francesca Di Rosa

**Affiliations:** ^1^Institute of Molecular Biology and Pathology, Consiglio Nazionale delle Ricerche, c/o Department of Molecular Medicine, Sapienza University, Rome, Italy

**Keywords:** immunological memory, CD8 T cells, bone marrow, homeostatic proliferation, bromodeoxyuridine

Several studies have shown that the bone marrow (BM) is implicated in the long-lasting persistence of memory CD8 T cells [see Ref. (1) and references therein]. Generally, it has been thought that the BM accomplishes this by sustaining a higher level of homeostatic proliferation of recirculating memory CD8 T cells than do spleen and lymph nodes (LN) in the steady state. This slow intermittent cell division would counteract cell death, thus contributing to the stable maintenance of memory T cell numbers over time. In a recent article entitled “Memory CD8^+^ T cells colocalize with IL-7^+^ stromal cells in bone marrow and rest in terms of proliferation and transcription,” Sercan Alp and coworkers challenge this view (2). They emphasize that results on memory CD8 T cell proliferation are discrepant and propose that the BM instead provides survival signals for resident memory CD8 T cells, as it does for plasma cells (3–5). They show that BM memory CD8 T cells colocalize with stromal cells, expressing the prosurvival cytokine IL-7. Moreover, they demonstrate that CD69, i.e., a typical marker of tissue-resident memory T cells, is expressed by a higher proportion of memory CD8 T cells in the BM than in the spleen. Finally, they show that freshly isolated BM memory CD8 T cells have a predominant resting transcriptional profile, in comparison with *in vitro*-activated CD8 T cells (2).

Starting from the article by Sercan Alp et al., this commentary revisits the data published so far on memory CD8 T cell proliferation in the BM and suggests that apparent discrepancies can be reconciled by a detailed analysis (see Table 1 and references therein). In respect to the interplay between memory CD8 T cells and other cells within BM niches and the possibility that BM memory T cells represent a pool of tissue-resident memory T cells, the reader is referred to another article in this issue (6).

**Table 1:** **Summary of published results on total CD8 and memory CD8 T cell proliferation in bone marrow, grouped according to experimental methods**.

	CD8 T cells	BM	Lymphoid periphery/blood	Reference
**(A) DNA content**				
**Species**				
Mouse	CD8^+^	↑	Spleen and PLN	Parretta et al. (7)
Mouse	CD8^+^ Ag-specific P14 (LCMV)	↑	Spleen, total LN, and blood	Becker et al. (8)
Mouse	CD8^+^ CD44^hi^	↑	Spleen	Sercan Alp et al. (2)
Human	CD8^+^ CD45RA^−^ CD45R0^+^	↑	Blood	Okhrimenko et al. (9)
**(B) BrdU**				
**BrdU administration**				
1 i.v. injection, 1 h before analysis	CD8^+^	↑	Spleen, PLN, MLN, CLN, and blood	Westermann et al. (10)
In drinking water for 3 d	CD8^+^	↑	Spleen, PLN, and MLN	Parretta et al. (7)
	CD8^+^ CD44^hi^	↑	Spleen, PLN, and MLN	
	CD8^+^ Ag-specific (OVA)	↑	Spleen and PLN	
	CD8^+^ Ag-specific OT-I (OVA)	↑	Spleen and PLN	
1 i.v. injection, 1 d before analysis	CD8^+^ CD44^hi^	↑	Spleen	Becker et al. (8)
	CD8^+^ Ag-specific P14 (LCMV)	↑	Spleen, total LN, and blood	
In drinking water for 3 d	CD8^+^ CD122^hi^	↑	Spleen, PLN, and MLN	Cassese et al. (11)
In drinking water for 3 d (tx mice)	CD8^+^	↑	Spleen and PLN	Parretta et al. (12)
In drinking water for either 14 or 42 d (tx mice)	CD8^+^ CD44^hi^	↑	Spleen and PLN	Parretta et al. (12)
In drinking water for either 5 or 8 d	CD8^+^	↑	Spleen	Snell et al. (13)
In drinking water with sugar for 3 d	CD8^+^ CD44^hi^	↑^a^	Spleen	Sercan Alp et al. (2)
**(C) CFSE**				
**CFSE-labeled cell transfer**				
Splenocyte transfer, either 15 or 25 d before analysis	CD8^+^ CD44^hi^	↑	Spleen, total LN, and blood	Becker et al. (8)
	CD8^+^ Ag-specific P14 (LCMV)	↑	Spleen, total LN, and blood	
Splenic CD8^+^ CD44^hi^ cell transfer, 7 d before analysis	CD8^+^ CD44^hi^	↑	Spleen and PLN	Quinci et al. (14)
*In vitro* primed OT-I splenocyte transfer, 21 d before analysis	CD8^+^ Ag-specific OT-I (OVA)	↑	Spleen and PLN	Lin et al. (15)

*The table summarizes published proliferation results on total CD8 and memory CD8 T cells in BM, in comparison with corresponding cells from lymphoid periphery/blood. The arrow ↑ indicates that results in BM were higher than those in lymphoid periphery/blood. Lymphoid periphery comprised spleen and LN, as indicated. Please, note that in all reports, spleen, LN, and blood were all concordantly lower than the BM. All results were obtained by flow cytometry, except for Westermann et al. (10), in which cell suspension analysis was performed by microscopy. (A) DNA content results are expressed as percentages of cells having 2*n* < DNA ≤ 4*n*; (B) BrdU results are expressed as percentages of BrdU^+^ cells after a BrdU pulse or BrdU continuous labeling in drinking water, as indicated; and (C) CFSE results are expressed as percentages of donor CFSE^+^ cells with decreased CFSE staining intensity, after CFSE-labeled cell transfer, as indicated. All BrdU and CFSE experiments were performed in mice, except for experiments by Westermann et al. (10), which were performed in rats*.

*Ag, antigen; BM, bone marrow; BrdU, bromodeoxyuridine; CFSE, carboxyfluorescein diacetate succinimidyl ester; CLN, cervical lymph nodes; d, day; h, hour; i.v., intravenous; LCMV, lymphochoriomeningitis virus; LN, lymph nodes; MLN, mesenteric lymph nodes; OT-I, TCR transgenic cells expressing a TCR against OVA; OVA, ovalbumin; PLN, peripheral lymph nodes; P14, TCR transgenic cells expressing a TCR against LCMV; Ref., reference; tx, thymectomized*.

*^a^Indicates that an abnormal proliferation was observed upon BrdU treatment (see text for details)*.

Sercan Alp and coworkers examined memory CD8 T cell proliferation or quiescence in mice by three methods, i.e., DNA content analysis, bromodeoxyuridine (BrdU) incorporation, and Ki67 staining (2).

DNA content analysis measures the percentage of cells in S/G_2_/M phase of cell cycle at a given time, thus providing a static index of proliferation in untreated individuals (16). By this method, Sercan Alp et al. found that the frequency of dividing cells within memory-phenotype CD44^high^ CD8 T cells in the BM was only about 0.4%. However, this low percentage was still three to eight times higher than that found in corresponding spleen samples [(2), BM 0.32–0.47% and spleen 0.05–0.10%; see Figures 4E and S3], in line with what has been seen in other studies by comparing CD8 T cells from BM with those from blood, spleen, or LN (7–9).

Sercan Alp et al. showed that assessment of CD8 T cell proliferation by BrdU incorporation may be misleading (2). BrdU is a thymidine analog that labels cells during S phase, thus marking the cells undergoing division in the course of BrdU treatment. Depending on dose and length of treatment, BrdU may have toxic effects, potentially leading to artifacts (17). In mice, BrdU is either injected or administered in drinking water, sometimes with sugar addition, a stratagem used to overcome the unpleasant taste of the analog (18, 19). Sugar can increase water consumption, e.g. in 4 hours mice drink 0.5–1.5 ml tap water and 2–4 times more water containing 10% sugar (20). However, total water intake is not usually measured in BrdU experiments, leaving actual BrdU dose undetermined. In the study by Sercan Alp et al., the mice were treated with 1 mg/ml BrdU plus 10% sugar in drinking water for 3 days, and there was an artificial rise – especially in the BM – of dividing memory CD8 T cell frequency, as measured by a BrdU-independent method [(2), Figure 4E]. Based on these results, the authors suggest that previous studies had greatly overestimated the extent of memory CD8 T cell proliferation (2).

However, other authors have used lower doses of BrdU without sugar (7, 12, 21–23), and Parretta et al. found little difference in proliferation (when tested by a BrdU-independent assay) between mice treated with BrdU or not (12). In more details, to compare the two groups of mice, Parretta et al. measured CD8 T cell proliferation by carboxyfluorescein diacetate succinimidyl ester (CFSE), a cytoplasmic dye that is equally distributed between daughter cells upon division. They reported that the proportion of dividing (i.e., CFSE^low^) CD8 T cells in spleen, LN, and BM in response to PolyI:C injection was similar when mice were either untreated or treated with 0.8 mg/ml BrdU in drinking water for 3 days (12), a standard protocol (24). PolyI:C treatment might have masked the toxic effects of BrdU (12); nevertheless, the dose of BrdU plus sugar is a major difference between the Sercan Alp et al.’s study and those of other groups. Indeed, the percentage of BrdU^+^ cells within spleen CD44^high^ CD8 T cells reported by Sercan Alp et al., i.e., about 30%, was definitely higher in comparison with previous data reported by several authors, all obtained with 0.8 mg/ml BrdU in drinking water for 3 days and no sugar (7, 21, 23). For example, Parretta et al. found that the fraction of BrdU^+^ cells within CD44^high^ CD8 T cells was 6% in spleen, 5% in LN, and 13% in BM, on average (7). Taking everything into account, it could be argued that the confounding effect of BrdU on BM CD8 T cell proliferation was dose dependent and limited to the study by Sercan Alp and coworkers (2).

Finally, Sercan Alp et al. analyzed CD8 T cells by intracellular staining for Ki67, a cell-cycle marker. They showed that on average, 93–95% of the memory CD8 T cells are negative for Ki67 (i.e., in G_0_ phase) in the BM and 88–94% in the spleen [(2), Figures 4B,D]. This indicates that the vast majority of the cells are quiescent and non-dividing at a given time, with a slight difference between BM and spleen. However, it should not be overlooked that the Ki67 assay does not give any information on frequency of dividing cells (i.e., in S/G_2_/M), since all cells in G_1_/S/G_2_/M score positively for Ki67. It appears that rather than being in contrast with previous findings on proliferation obtained by other methods (see Table 1 and references therein), the Ki67 results in the Sercan Alp et al.’s study simply report on a different aspect of the cell cycle.

Table 1 is a summary of published findings on total CD8 and memory CD8 T cell proliferation in BM, grouped according to the experimental methods. In addition to DNA content and BrdU, some authors used CFSE. For example, Quinci et al. found that in 1 week the fraction of CFSE^low^ cells within CD44^high^ CD8 T cells was 17% in spleen, 17% in LN, and 27% in BM, on average (14). All data in Table 1 show a higher percentage of proliferating cells within memory CD8 T cells in the BM than in lymphoid periphery, i.e., spleen, LN, and blood.

Thus, the data on proliferation are in agreement, while discrepancies remain in interpretations (25, 26). The main point of contention is how much the proliferation occurring in the BM contributes to the long-term maintenance of memory CD8 T cells. Sercan Alp et al. focused their attention on the paucity of proliferating cells in their BM samples (2), ignoring that this is, nevertheless, a higher proportion than that found in spleen and LN. It could be argued that such difference in proliferating cell frequencies should not be neglected, in light of the fact that BM memory CD8 T cells are a large population. Indeed, the BM is a huge organ and, moreover, after the peak of an acute response, antigen-specific CD8 T cell contraction is often less pronounced in the BM than in other organs, resulting in a high number of memory CD8 T cells in the BM in the memory phase (7, 8, 27, 28). For example, in the contraction phase of the response against the M-45 epitope of murine cytomegalovirus (MCMV), the frequency of antigen-specific CD8 T cells dropped 14–20 times in the blood, liver, and lung, and only about five times in the BM (28). Moreover, at late times (6–10 weeks) after immunization against the model antigen ovalbumin, the number of antigen-specific memory CD8 T cells in the BM was 2–3 times higher than that in the spleen and 3–11 times higher than that in total LN (7). However, enrichment of antigen-specific CD8 T cells in the BM in the memory phase was not observed in other types of responses. For example, at late times after infection, antigen-specific memory CD8 T cell frequency in the BM was not higher than that in blood, liver, or lung in the inflationary response against the M-38 epitope of MCMV (28) or in the response against vaccinia virus induced by skin scarification, which mostly elicited antigen-specific tissue-resident memory CD8 T cells in the skin (29).

Taking everything into account, the BM plays a preferential role in sustaining the homeostatic proliferation of antigen-specific memory CD8 T cells following classical acute responses resulting in the long-term systemic memory (1, 7, 8, 30).

## Author Contributions

The author confirms being the sole contributor of this work and approved it for publication.

## Conflict of Interest Statement

The author declares that the research was conducted in the absence of any commercial or financial relationships that could be construed as a potential conflict of interest.
